# Incidence and Outcome of Dengue Fever During Pre-monsoon, Monsoon, and Post-monsoon Periods: A Cross-Sectional Study From a Tertiary Care Hospital in India

**DOI:** 10.7759/cureus.82669

**Published:** 2025-04-21

**Authors:** Swastik Acharya, Shilpa Mishra, Arushi Choudhary, Shubham Desale, Vibha Sharma, Shubhransu Patro

**Affiliations:** 1 Department of General Medicine, DRIEMS Institute of Health Sciences and Padmini Care Hospital, Tangi, IND; 2 Department of Obstetrics and Gynecology, DRIEMS Institute of Health Sciences and Padmini Care Hospital, Tangi, IND; 3 Department of General Medicine, Kalinga Institute of Medical Sciences, Bhubaneswar, IND

**Keywords:** acute kidney injury, capillary leakage, correlation, dengue, monsoon, ns1 antigen, platelet, transfusion

## Abstract

Background and objectives: Lately, dengue fever has emerged as a public health concern in India. Overcrowding and climate change facilitated this vector-borne disease. Hence, we carried out this study to evaluate the incidence and outcome of dengue fever in monsoon, post-monsoon, and pre-monsoon periods. Additionally, we correlated the age, platelet count, and duration of hospitalization of the participants.

Methods: We conducted this cross-sectional study from June 2019 to September 2021 at Kalinga Institute of Medical Sciences (KIMS), Bhubaneswar, India. We obtained data (age, gender, organs affected, platelet count, requirement of ICU, platelet transfusion, duration of hospitalization, and outcome) from their case records. R software version 4.1.3 (R Foundation for Statistical Computing, Vienna, Austria) was used for data analysis.

Results: Seven hundred eighteen dengue patients were recruited for this study. Their mean age was 43.2 ± 11.8 years. There were 347 (48.3%) female participants. The mean platelet count of the study population was 132.1 ± 64.7 × 10^9^/L. The dengue cases were the highest (561, 78.1%) during the monsoon period. Most participants had their kidneys (185, 25.8%) affected due to dengue fever. A total of 224 (31.2%) patients had bleeding manifestations, and 371 (51.7%) patients required ICU admission. The average duration of hospital stay was 11.0 ± 3.9 days. Of 718 patients, 693 (96.5%) were discharged and 25 (3.5%) died. The platelet count and duration of hospital stay were negatively correlated (r = -0.390, p < 0.001).

Conclusion: Dengue cases were maximum during the monsoon period. Younger individuals and men were more affected. Kidney involvement was the maximum, followed by lungs, liver, and heart. The duration of hospitalization and platelet count were inversely related.

## Introduction

In India, dengue has become one of the most common vector-borne infectious diseases [1-3]. It might be associated with seasonal variation and overcrowding in India [3,4]. After the 1990s, malaria incidence in India was gradually reduced [5]. Furthermore, dengue cases increased in number. For the last three decades, dengue has posed a significant public health concern in India [3,6].

Dengue is transmitted to human beings through *Aedes aegypti* and *Aedes albopictus* [7,8]. Although the virus and its vector are active throughout the year in India, dengue fever has the highest occurrence during monsoon periods [9]. Rainy or post-rainy seasons allow water accumulation in coconut shells, coolers, old tires, and ancient earthenware pots. These items serve as a potential breeding ground for mosquitoes [9,10]. As a result, the vector grows, and dengue incidence rises [9]. There are more mosquitoes (3-4 females per house) during the rainy season than there are during the dry season (1-2 females per house) [11]. Dengue fever exhibits cyclical and seasonal patterns in India, with significant outbreaks every 2-3 years [9-11].

Acute kidney injury (AKI) is considered one of the most serious complications of dengue [12]. A sharp reduction in the glomerular filtration rate characterizes it. In the last three decades, the frequency of kidney damage has dramatically increased in patients with dengue fever [12,13]. Hypotension, direct damage due to the virus, immune system-mediated indirect mechanisms, and rhabdomyolysis could lead to AKI [12,13]. Patients with dengue frequently have liver damage. Liver enzymes are temporarily mildly elevated in most dengue patients [14,15]. A condition known as dengue-induced severe hepatitis (DISH) occurs in 4%-15% of dengue patients [14]. Transaminase levels are increased around 10 times in patients with DISH [14]. Thrombocytopenia, hemorrhage, and increased capillary permeability may lead to respiratory problems such as pleural effusion, pneumonitis, and acute respiratory distress syndrome (ARDS) [16]. Oxidative stress and increased viral load may trigger myocarditis and heart failure [17,18]. Thrombocytopenia in dengue fever leads to bleeding manifestations. Patients with platelet values below 50,000/mm^3^ with bleeding symptoms are managed with random donor platelet (RDP) transfusion [19]. Nevertheless, RDP transfusion becomes mandatory for those with critically low levels of platelets (<10,000/mm^3^) even without bleeding symptoms [19,20].

In India, the monsoon period spans from June to September. The next four months, i.e., October to January, are considered post-monsoon. The remaining four months, i.e., February to May, are pre-monsoon periods [21]. The incidence of dengue fever, duration of hospitalization, and outcome of patients are affected by numerous factors such as socioeconomic status, accessibility to healthcare, and comorbidities. We carried out this investigation to determine the incidence of dengue fever in India during the monsoon, post-monsoon, and pre-monsoon seasons. Additionally, we assessed the patients' seropositivity, gender-specific parameter variations, and correlations between age, platelet count, and duration of hospitalization.

## Materials and methods

This cross-sectional study was conducted from June 18, 2019, to September 30, 2021. Our study was approved by the Institutional Ethics Committee of Kalinga Institute of Medical Sciences (KIMS) (approval number: KIIT/KIMS/IEC/18/2019). We followed the Good Clinical Practices and the Declaration of Helsinki.

Study criteria

In this study, we recruited adult patients admitted to our hospital with dengue fever within the specified time frame. We excluded patients below 18 years of age, patients with bleeding disorders, and pregnant women.

Study procedure

We obtained the data of eligible participants from their case records. We noted their age, gender, platelet counts, and comorbidities such as diabetes and hypertension. We also recorded the organs affected at admission or during their hospital stays. We mentioned the month, season, and year of the hospital admission. We denoted the following months as per the monsoon phase in India: June to September (monsoon period), October to January (post-monsoon period), and February to May (pre-monsoon period). The positivity status of serum NS1 Ag, IgM Ab, and IgG Ab of all participants was noted. We recorded the requirement for RDP transfusion, ICU admission, and duration of hospitalization.

Statistical analysis

Eligible participants were recruited through convenience sampling. Continuous variables were shown as mean and standard deviation. Categorical variables were shown as numbers and percentages. We analyzed the participants' age distribution and platelet counts with jitter plots. Organ involvement and other parameters (e.g., ICU entry and RDP transfusion) during various seasons were assessed with pie diagrams. We evaluated the associations among age, platelet count, and duration of hospitalization with correlation plots. R software version 4.1.3 (R Foundation for Statistical Computing, Vienna, Austria) was used [22]. A p-value of 0.05 or less demonstrates statistical significance. We assessed categorical variables with the Chi-square test. Continuous variables were assessed through the unpaired t-test or ANOVA. Associations were analyzed using Pearson's correlation.

## Results

We recruited 718 patients with dengue fever from June 18, 2019, to September 30, 2021. We divided the calendar year into the following three seasons as per the monsoon period in Eastern India: monsoon (June to September), post-monsoon (October to January), and pre-monsoon (February to May). The incidence of dengue fever in our hospital during the study period is shown in Table 1. The highest number of dengue cases was seen during the monsoon period (total: 561 cases, 201 in 2019, 165 in 2020, and 195 in 2021). The number of female patients was higher in 2019. In 2020 and 2021, there were increased incidences of dengue fever in male patients compared to female patients. During the study period, 371 (51.7%) male and 347 (48.3%) female patients suffered from dengue fever.

**Table 1:** Incidence of dengue fever during the study period Numbers show the incidence of dengue fever in male and female patients during the study period (June 18, 2019, to September 30, 2021).

Time		2019			2020			2021		
		Total (n = 239)	Male (n = 107)	Female (n = 132)	Total (n = 244)	Male (n = 136)	Female (n = 108)	Total (n = 235)	Male (n = 128)	Female (n = 107)
Monsoon	June	24 (10.0%)	12 (11.2%)	12 (9.1%)	11 (4.5%)	6 (4.4%)	5 (4.6%)	37 (15.7%)	19 (14.8%)	18 (16.8%)
	July	47 (19.7%)	20 (18.7%)	27 (20.4%)	43 (17.6%)	23 (16.9%)	20 (18.5%)	55 (23.4%)	31 (24.2%)	24 (22.4%)
	August	73 (30.5%)	33 (30.8%)	40 (30.3%)	65 (26.6%)	36 (26.5%)	29 (26.9%)	61 (26.0%)	34 (26.6%)	27 (25.2%)
	September	57 (23.8%)	26 (24.3%)	31 (23.5%)	46 (18.9%)	26 (19.1%)	20 (18.5%)	42 (17.9%)	23 (18.0%)	19 (17.8%)
Post-monsoon	October	15 (6.3%)	7 (6.5%)	8 (6.1%)	20 (8.2%)	11 (8.1%)	9 (8.3%)	0	0	0
	November	13 (5.4%)	6 (5.6%)	7 (5.3%)	14 (5.7%)	7 (5.1%)	7 (6.5%)	0	0	0
	December	10 (4.2%)	3 (2.8%)	7 (5.3%)	12 (4.9%)	7 (5.1%)	5 (4.6%)	0	0	0
	January	0	0	0	10 (4.1%)	7 (5.1%)	3 (2.8%)	12 (5.1%)	6 (4.7%)	6 (5.6%)
Pre-monsoon	February	0	0	0	7 (2.9%)	4 (2.9%)	3 (2.8%)	6 (2.6%)	3 (2.3%)	3 (2.8%)
	March	0	0	0	6 (2.5%)	2 (1.5%)	4 (3.7%)	8 (3.4%)	4 (3.1%)	4 (3.7%)
	April	0	0	0	5 (2.0%)	3 (2.2%)	2 (1.9%)	5 (2.1%)	4 (3.1%)	1 (0.9%)
	May	0	0	0	5 (2.0%)	4 (2.9%)	1 (0.9%)	9 (3.8%)	4 (3.1%)	5 (4.7%)

The demographics of the 718 patients are shown in Table 2. The average age of the patients was 43.2 ± 11.8 years. A total of 347 (48.3%) participants were females. Diabetes and hypertension were present among 220 (30.6%) and 80 (11.1%), respectively. The most common organ involved during hospitalization was the kidney (185, 25.8%), followed by the liver (40, 5.6%), lungs (40, 5.6%), and heart (15, 2.1%). Moreover, 224 (31.2%) patients had bleeding manifestations. The mean platelet count during admission was 132.1 ± 64.7 × 10^9^/L. Overall, 371 (51.7%) patients required ICU admission. The average duration of hospital stay was 11.0 ± 3.9 days. Of the 718 patients, 25 (3.5%) died during their hospital stay.

**Table 2:** Demographics of the study population Categorical data are shown as numbers and percentages. Continuous data are shown as means and standard deviations. Continuous and categorical variables were assessed with the unpaired t-test and Chi-square test, respectively. RDP: random donor platelet, ICU: intensive care unit

Parameters	Total (N = 718)	Male (n = 371)	Female (n = 347)	Statistics	p-value
Age in years	43.2 ± 11.8	42.8 ± 12.2	43.6 ± 11.4	98.1	<0.001
Age group					
19-40 years	315 (43.9%)	172 (46.4%)	143 (41.2%)	2312.7	<0.001
41-60 years	346 (48.2%)	170 (45.8%)	176 (50.7%)		
60 years	57 (7.9%)	29 (7.8%)	28 (8.1%)		
Diabetes	220 (30.6%)	114 (30.7%)	106 (30.5%)	11.4	0.04
Hypertension	80 (11.1%)	45 (12.1%)	35 (10.1%)	10.8	0.04
Organ involvement					
Kidney	185 (25.8%)	99 (26.7%)	86 (24.8%)	37.6	<0.001
Liver	40 (5.6%)	29 (7.8%)	11 (3.2%)	123.8	<0.001
Lungs	40 (5.6%)	21 (5.7%)	19 (5.5%)	13.6	0.02
Heart	15 (2.1%)	9 (2.4%)	6 (1.7%)	41.4	<0.001
Bleeding symptoms	224 (31.2%)	125 (33.7%)	99 (28.5%)	112.9	<0.001
Platelet counts (10^9^/L)	132.1 ± 64.7	128.6 ± 64.9	135.9 ± 64.4	91.7	<0.001
RDP transfusion	224 (31.2%)	125 (33.7%)	99 (28.5%)	167.5	<0.001
ICU requirement	371 (51.7%)	203 (54.7%)	168 (48.4%)	257.3	<0.001
Hospital stays in days	11.0 ± 3.9	11.2 ± 3.8	10.9 ± 4.2	11.1	0.04
Discharge	693 (96.5%)	354 (95.4%)	339 (97.7%)	1882.1	<0.001
Death	25 (3.5%)	17 (4.6%)	8 (2.3%)		

The seropositivity of the 718 patients is shown in Table 3. Overall, 303 (42.2%) had only positive serum NS1 Ag during admission. A total of 75 (10.4%) patients had positive reports for serum NS1 Ag, IgM Ab, and IgG Ab. The statistically significant differences could be attributed to the seasonal variation of dengue cases.

**Table 3:** Seropositivity of the study population Categorical data are shown as numbers and percentages. Categorical variables were assessed using the Chi-square test. NS1 Ag: dengue non-structural protein 1 antigen

Serological markers	Total (n = 718)	Monsoon period (n = 561)	Post-monsoon period (n = 106)	Pre-monsoon period (n = 51)	Statistics	p-value
Only NS1 Ag positive	303 (42.2%)	256 (45.6%)	28 (26.4%)	19 (37.2%)	1722.5	<0.001
Only IgM Ab positive	40 (5.6%)	28 (5.0%)	9 (8.5%)	3 (5.9%)	317.8	<0.001
Only IgG Ab positive	102 (14.2%)	77 (13.7%)	20 (18.8%)	5 (9.8%)	743.9	<0.001
NS1 Ag and IgM Ab positive	45 (6.3%)	26 (4.6%)	13 (12.3%)	6 (11.8%)	148.3	<0.001
NS1 Ag and IgG Ab positive	19 (2.6%)	11 (2.0%)	6 (5.7%)	2 (3.9%)	92.1	<0.001
IgM Ab and IgG Ab positive	134 (18.7%)	113 (20.1%)	11 (10.4%)	10 (19.6%)	396.4	<0.001
NS1 Ag, IgM Ab, and IgG Ab positive	75 (10.4%)	50 (9.0%)	19 (17.9%)	6 (11.8%)	1102.6	<0.001

**Figure 1:**
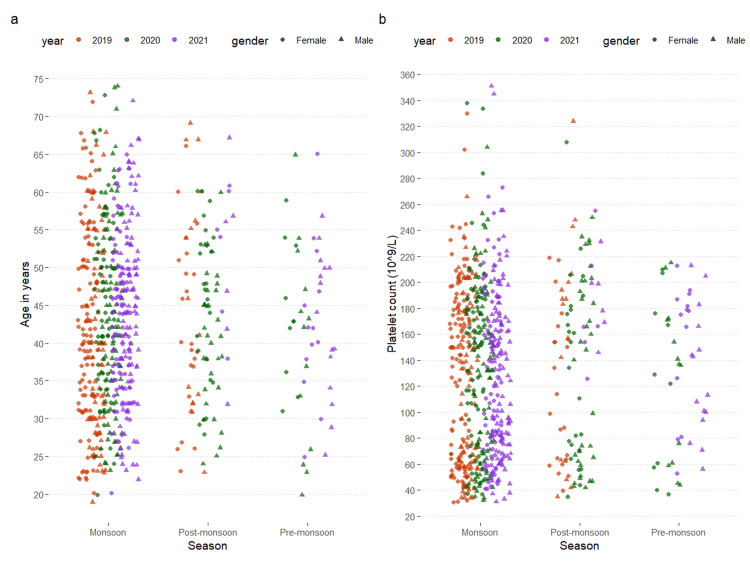
Age distribution and platelet counts of the participants The jitter plots show the age distribution and platelet counts of the participants. The calendar years (2019, 2020, and 2021) and genders (male and female) were expressed in different colors and shapes, respectively. The age distribution of all participants is shown in Figure 1a. The mean platelet count of all participants at the time of admission is shown in Figure 1b.

The age distribution of the participants is shown in Figure 1a. The highest number of patients (560) had dengue fever during the monsoon period (from June to September). The mean ages of patients admitted during the monsoon, post-monsoon, and pre-monsoon periods were 43.1 ± 11.9 years, 44.0 ± 11.4 years, and 42.0 ± 10.8 years, respectively. The difference was statistically significant (p < 0.001). The platelet counts of the participants are shown in Figure 1b. The mean platelet counts of patients admitted during the monsoon, post-monsoon, and pre-monsoon periods were 130.7 ± 64.4 × 10^9^/L, 139.2 ± 70.0 × 10^9^/L, and 132.4 ± 56.6 × 10^9^/L, respectively. The difference was statistically significant (p < 0.001).

**Figure 2:**
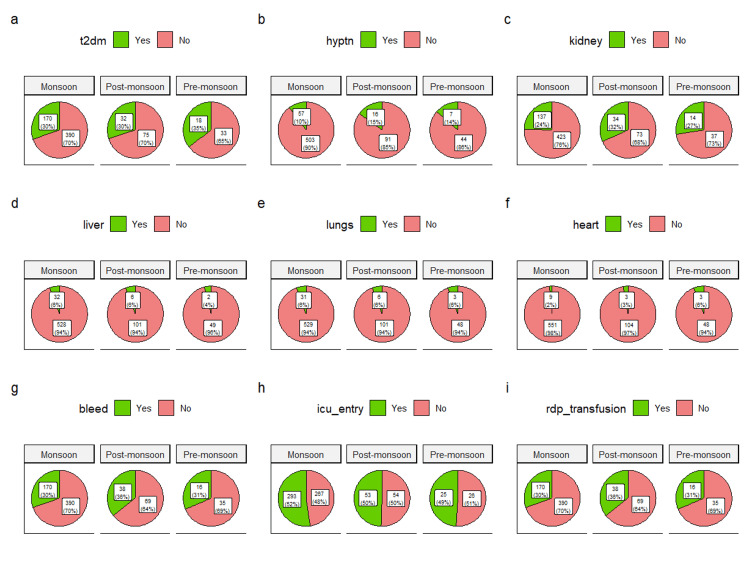
Variations of parameters according to various seasons The pie diagrams illustrate the variations of parameters in different seasons during the study period. Among the participants admitted during various periods, Figure 2a shows the prevalence of diabetes, Figure 2b shows the prevalence of hypertension, Figure 2c shows the proportion of patients with renal involvement, and Figure 2d shows the proportion of patients with hepatic involvement. Figure 2e shows the proportion of patients with involvement of the respiratory system, Figure 2f shows the proportion of patients with involvement of the heart, Figure 2g shows the proportion of patients with bleeding symptoms, Figure 2h shows the proportion of patients admitted to the ICU, and Figure 2i shows the proportion of patients who required RDP transfusion. T2dm: type 2 diabetes mellitus, hyptn: hypertension, rdp: random donor platelet, ICU: intensive care unit

The comorbidities, organ involvement, ICU admission, and RDP transfusion of the study participants are illustrated through pie diagrams in Figure 2. All nine plots shown in Figure 2a-2i illustrate the variations of parameters in different seasons. Of the 718 participants, 220 (30.6%) were diabetic (Figure 2a), and 80 (11.1%) were hypertensive (Figure 2b). Kidney involvement was seen among 185 (25.8%) patients (Figure 2c). The liver was affected in 40 (5.6%) patients (Figure 2d). Lungs were affected in 40 (5.6%) patients (Figure 2e). Fifteen (2.1%) patients had heart involvement (Figure 2f). Bleeding manifestations were seen among 224 (31.2%) patients (Figure 2g). Of the 718 patients, 371 (51.7%) were admitted to the ICU (Figure 2h). RDP was transfused to 224 (31.2%) patients (Figure 2i).

**Figure 3:**
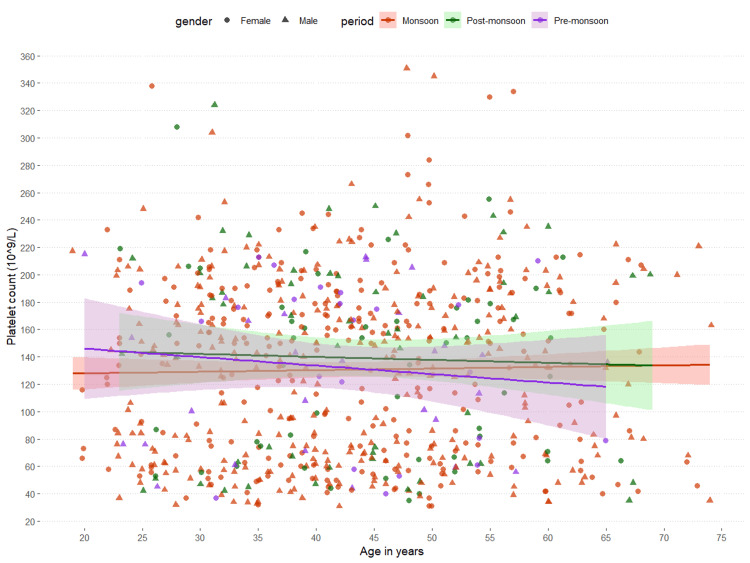
Association between age and platelet count The scatterplot shows the association between age and platelet counts of the participants. The periods (monsoon, post-monsoon, and pre-monsoon) and genders (male and female) were expressed in different colors and shapes, respectively. We analyzed the association with Pearson's correlation test. The correlation coefficients and corresponding p-values are as follows: monsoon: r = 0.021, p = 0.61; post-monsoon: r = -0.035, p = 0.72; and pre-monsoon: r = -0.117, p = 0.41.

The association between the age of the patients and their platelet counts during hospital admission is shown in Figure 3. The x and y axes of the scatterplot represent the age in years and platelet counts, respectively. There was a weakly positive association between the age of the patients and their platelet counts during hospital admission (r = 0.006, p = 0.87). The associations were similar for all three periods (monsoon: r = 0.021, p = 0.61; post-monsoon: r = -0.035, p = 0.72; pre-monsoon: r = -0.117, p = 0.41). The association between the age of the patients and their platelet counts during hospital admission was found to be non-significant.

**Figure 4:**
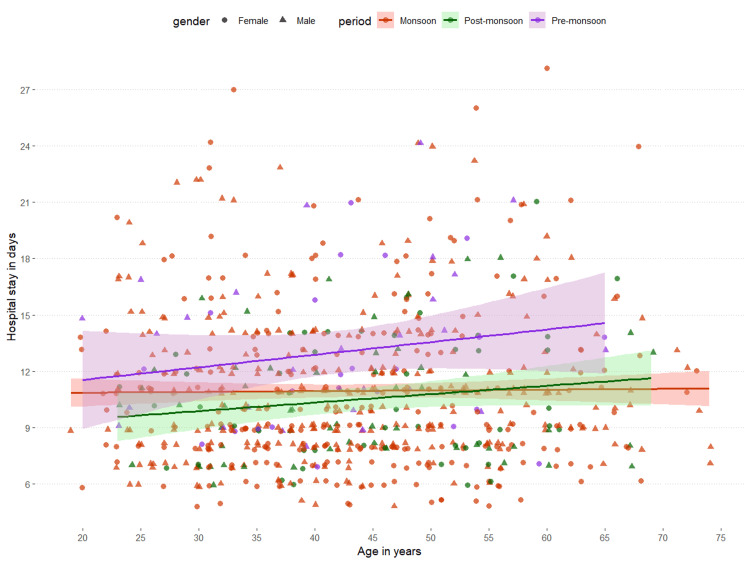
Association between age and hospital stay The scatterplot shows the association between age and the duration of hospital stay of the participants. The periods (monsoon, post-monsoon, and pre-monsoon) and genders (male and female) were expressed in different colors and shapes, respectively. We analyzed the association with Pearson's correlation test. The correlation coefficients and corresponding p-values are as follows: monsoon: r = 0.012, p = 0.77; post-monsoon: r = 0.160, p = 0.10; pre-monsoon: r = 0.178, p = 0.21.

The association between the age of the patients and their duration of hospital stay is shown in Figure 4. The x and y axes of the scatterplot represent the age (in years) and duration of hospital stay (in days), respectively. There was a weakly positive association between the age of the patients and their duration of hospital stay (r = 0.035, p = 0.35). The associations were similar for all three periods (monsoon: r = 0.012, p = 0.77; post-monsoon: r = 0.160, p = 0.10; pre-monsoon: r = 0.178, p = 0.21). The association between the age of the patients and their duration of hospital stay was found to be non-significant.

**Figure 5:**
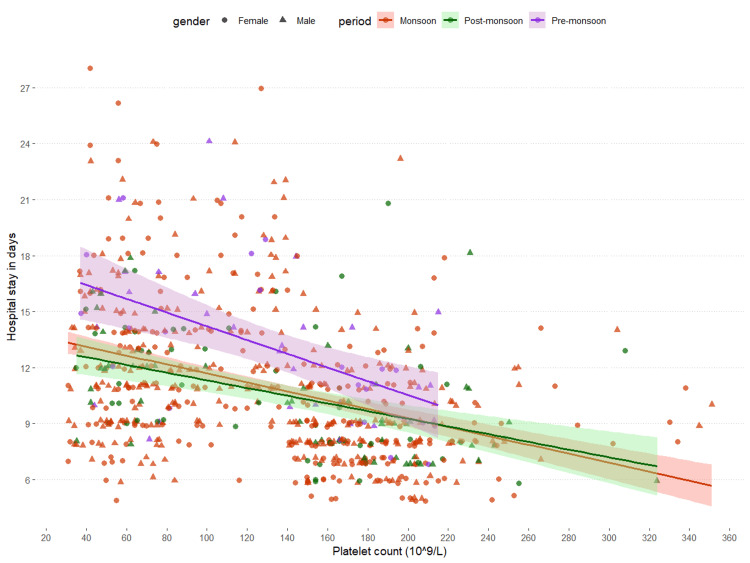
Association between platelet count and hospital stay The scatterplot shows the association between platelet count and the duration of hospital stay of the participants. The periods (monsoon, post-monsoon, and pre-monsoon) and genders (male and female) were expressed in different colors and shapes, respectively. We analyzed the association with Pearson's correlation test. The correlation coefficients and corresponding p-values are as follows: monsoon: r = -0.378, p < 0.001; post-monsoon: r = -0.450, p < 0.001; pre-monsoon: r = -0.515, p < 0.001.

The association between the platelet counts during hospital admission and durations of hospital stay is shown in Figure 5. The x and y axes of the scatterplot represent the platelet counts and durations of hospital stay (in days), respectively. There was a negative association between platelet counts during hospital admission and durations of hospital stay (r = -0.390, p < 0.001). The associations were similar for all three periods (monsoon: r = -0.378, p < 0.001; post-monsoon: r = -0.450, p < 0.001; pre-monsoon: r = -0.515, p < 0.001). The negative association between platelet counts during hospital admission and durations of hospital stay was found to be significant. The findings suggest that patients with a low platelet count were prone to prolonged hospitalization.

## Discussion

We analyzed the data of 718 dengue patients in this study. Of them, 561 (78.1%) were admitted during the monsoon period. Dengue cases during the post-monsoon and pre-monsoon periods were 106 (14.8%) and 51 (7.1%), respectively. The mean age of the participants was 43.2 ± 11.8 years. There was a male predominance in our study. Overall, 371 (51.7%) participants were males. The mean platelet count of the participants was 132.1 ± 64.7 × 10^9^/L. The demographic data matched two recently conducted studies [3,8]. A total of 220 (30.6%) subjects were diabetic, and 80 (11.1%) were hypertensive. The kidney was the most common organ to be affected, followed by the liver, lungs, and heart. Bleeding manifestations were seen in 224 (31.2%) subjects. A total of 371 (51.7%) patients were admitted to the ICU during their hospital stays. The mean duration of hospitalization was 11.0 ± 3.9 days. Our findings matched the studies by Mallhi et al. [23] and Ho et al. [24]. Moreover, 693 (96.5%) participants were discharged after successful management of dengue fever, and the remaining 25 (3.5%) subjects died.

Tropical countries like India face substantial climate change throughout the year. Rainy season, enormous population, inadequate drainage system, and overcrowding all together facilitate perennial growth of mosquitoes. During the monsoon period, the mosquito population rises, as well as the diseases transmitted by them (e.g., dengue, malaria) [25,26]. Younger individuals were more commonly affected than older ones. Male subjects predominated our study population. These observations matched previous studies [3,9,27]. The majority of our participants had a low platelet count during admission. A total of 371 participants had bleeding symptoms and required RDP transfusion. Capillary leakage and thrombocytopenia trigger the bleeding manifestations [3,8,19]. The subjects had seropositivity for one or more of the following: serum NS1 Ag, IgM Ab, and IgG Ab. The highest positivity (303, 42.2%) was seen for serum NS1 Ag.

The most common organ affected in our study population was the kidney. AKI might be triggered by increased viral load, capillary leakage, immune-mediated reactions, or rhabdomyolysis [12,13]. The liver, lungs, and heart were the other organs affected. Participant age was weakly associated with platelet count and duration of hospitalization. However, the duration of hospital stay was prolonged for those with low platelet counts during admission. Our observations matched a recently conducted study [28].

The main strength of our study was data analysis as per the seasonal variation in India. We also assessed the correlation among age, platelet count, and duration of hospitalization. Our study had a few limitations. The hospital admissions during the study period were significantly affected by the COVID-19 pandemic. Moreover, the discharge rates were also affected by the same. This had caused prolonged hospitalization for some patients. We did not evaluate the medications of the patients and laboratory parameters for the seasonal variations, as well as the quality of life of the patients.

## Conclusions

Dengue cases were maximum during the monsoon period. Younger individuals were more affected than older ones. Kidney involvement was the highest, followed by the liver, lungs, and heart. The platelet count and duration of hospitalization were inversely related. More prospective studies should be conducted with a larger sample size to assess the seasonal variation of dengue infection and its attributes.
